# Riboflavin as a Dual-Function Additive for Enhancing Biodegradation in Piezoelectric PLA/BT Composites

**DOI:** 10.3390/ma18163860

**Published:** 2025-08-18

**Authors:** Natalia Puszczykowska, Piotr Rytlewski, Agnieszka Mirkowska, Paweł Cyprys, Piotr Augustyn, Kacper Fiedurek

**Affiliations:** 1 Faculty of Materials Engineering, Kazimierz Wielki University, Chodkiewicza 30, 85-064 Bydgoszcz, Poland; prytlewski@ukw.edu.pl (P.R.); augustyn@ukw.edu.pl (P.A.); 2 Faculty of Electrical Engineering, Wroclaw University of Science and Technology, pl. Grunwaldzki 13, 50-377 Wroclaw, Poland; agnieszka.mirkowska@pwr.edu.pl; 3 Faculty of Mechatronics, Kazimierz Wielki University, Kopernika 1, 85-074 Bydgoszcz, Poland; pawcyp00@ukw.edu.pl (P.C.); kacfie95@ukw.edu.pl (K.F.)

**Keywords:** PLA, BaTiO_3_, riboflavin, biodegradation, piezoelectric biomaterials, piezoelectric composites

## Abstract

Poly(lactic acid)/barium titanate (PLA/BT) composites exhibit piezoelectric properties desirable for bone tissue engineering, but their low biodegradability limits implant resorption. In this study, riboflavin (RF) is introduced as a dual-function additive that enhances biodegradation in PLA/BT composites. Its addition led to significantly increased microbial colonization and a five-fold higher mass loss compared to unmodified samples. These observations are consistent with the known polarity of RF and its role as a cofactor in microbial metabolism. The PLA/BT/RF composites are subjected to full characterization, including thermogravimetric analysis (TG), differential scanning calorimetry (DSC), tensile testing, dynamic mechanical analysis (DMA), dielectric permittivity measurements, and determination of piezoelectric coefficient d_33_. Compared to PLA/BT, RF-containing composites exhibit significantly accelerated biodegradation, with mass loss reaching up to 16% after 28 days, while maintaining functional piezoelectricity (d_33_ ≈ 3.9 pC/N). Scanning electron microscopy (SEM) performed after biodegradation reveals intensified microbial colonization and surface deterioration in the RF-modified samples. The data confirm that riboflavin serves as an effective modifier, enabling controlled biodegradation without compromising electromechanical performance. These results support the use of PLA-based piezoelectric composites for resorbable biomedical implants.

## 1. Introduction

In recent years, increasing attention has been paid to functional materials designed for regenerative medicine [1,2]. The main challenges facing materials used in biomedical applications include especially their biodegradability and biocompatibility and potential for tissue regeneration. Piezoelectric biomaterials are gaining traction in tissue engineering, particularly in the context of bone regeneration [3,4,5,6]. These materials can stimulate osteoblast migration and differentiation, thereby supporting bone tissue mineralization [7,8,9]. Moreover, the piezoelectric effect itself enhances osteoblast activity and accelerates bone regeneration [3,10,11,12,13,14].

The piezoelectric effect occurs both in organic materials (naturally derived piezoelectric materials), such as peptides, wood, or gelatin, and in synthetic piezoelectric polymers such as poly(lactic acid), PLA, or polyhydroxybutyrate (PHB) [15,16,17]. Great emphasis is placed on the development of biodegradable polymer-based composites such as PLA/BT (barium titanate, BT) or PHB/ZNO (zinc oxide, ZnO) [18,19,20]. The main advance of such composites is that the electro-mechanical properties can be controlled by the addition of a precise amount of piezoelectric filler and proper processing [19]. Moreover, the PLA composites with BT show particular promise in implant applications, combining the flexibility of the polymer with the piezoelectric properties of the ceramic phase [18,21,22,23]. As a result, they can promote bone regeneration without the need for external stimulation or secondary surgery for implant removal [13,24,25,26].

Despite the numerous advantages of PLA as a biomaterial, its inherently slow biodegradation remains a significant limitation in implantology [27]. Under biological conditions, degradation may take months or even years, leading to prolonged retention of the implant after its function has been fulfilled [3,13,28]. This process occurs primarily through hydrolysis of ester bonds, with the rate depending on factors such as pH, temperature, and enzymatic activity [29].

Notably, the degradation mechanism of PLA remains the same across different biological environments—such as compost, microbial suspensions, or the human body—as it proceeds through ester bond hydrolysis. Variations in the degradation rate are primarily attributed to external conditions, including humidity, enzymatic activity, and the accessibility of water to the polymer matrix.

Attempts to address this issue include doping PLA with various additives such as mineral fillers (e.g., hydroxyapatite) and organic compounds that alter hydrophilicity or influence the microbiota. However, achieving a reliably accelerated degradation rate remains a challenge [30,31].

A promising approach involves doping PLA with riboflavin (RF). Incorporating RF into polymer composites—particularly those that also contain a ceramic phase like BT—can yield multifunctional materials that retain desirable mechanical and piezoelectric properties while exhibiting enhanced resorption in biological environments. Studies suggest that RF may promote PLA biodegradation, alter the microbial environment, and modify the surface properties of the composite [32].

Riboflavin is also a precursor of flavin adenine dinucleotide (FAD), a coenzyme essential to the tricarboxylic acid (TCA) cycle. Enhancing mitochondrial metabolism, it stimulates the proliferation and adhesion of tissue-forming cells such as osteoblasts and fibroblasts. Faster implant colonization shortens degradation time, reducing the risk of infection due to long-term material retention.

RF further demonstrates excellent biocompatibility with bone cells and may offer protection against oxidative stress during tissue regeneration. These features, combined with its antimicrobial properties, reinforce its potential in bone tissue engineering [33,34,35]. The ability to simultaneously accelerate PLA degradation and enhance regenerative functions makes riboflavin a highly attractive additive for such biomaterials.

From a clinical perspective, combining the piezoelectric effect with controlled biodegradation is highly desirable: an implant that both actively stimulates bone growth by generating electrical charges in response to mechanical load and gradually resorbs in sync with tissue healing eliminates the need for a second surgery to remove the scaffold or stabilizer [10,36,37]. Therefore, developing multifunctional composites such as PLA/BT/RF is essential for advancing bone-regenerative biomaterials.

This study aims to comprehensively characterize such materials by investigating the piezoelectric properties of the PLA/BT/RF composite and assessing the impact of riboflavin on its biodegradation. A comparative analysis is presented between PLA/BT and PLA/BT/RF composites, focusing on thermal stability, mechanical strength, piezoelectric properties (including d_33_ coefficient measurements), and degradation under laboratory conditions.

Taken together, these features confirm that riboflavin is a multifunctional additive that not only improves the bioactivity and degradability of PLA-based composites but also enhances implant safety by reducing infection risk. Its dual role in supporting both tissue integration and material resorption addresses two critical challenges in bone implant design.

## 2. Materials and Methods

### 2.1. Materials

The following materials were used in the study:

- Poly(lactic acid), (PLA), with 96% lactic acid L-isomer content, specific density of 1.24 g/cm^3^ and melt mass index of 3 g/10 min (190 °C, 2.16 kg), was supplied by TotalEnergies-Corbion (Gorinchem, The Netherlands). The material was ground to grains with an average size of 150–200 µm.
- Barium titanate (IV) (BaTiO_3_, BT) with a density of 6.08 g/cm^3^, particle size < 3 µm, was supplied by MERCK Poland (Poznań, Poland).
- Riboflavin (RF, vitamin B_2_), RF, with a molecular weight of 376.36 g/mol and a density of about 1.65 g/cm^3^, was provided by Food Colours (Piotrków Trybunalski, Poland).

### 2.2. Manufacturing of Composites

PLA, BT, and RF powders were dried at 70 °C for 6 h in a SLW-53 dryer (POL-EKO, Wodzislaw Slaski, Poland) and then mechanically mixed. The mixtures were prepared in 1 dm^3^ batches, according to the values given in Table 1.

The characteristics and processing parameters of PLA/BT composites are described in the article [19], while PLA/BT/RF composites were produced on a twin-screw extruder with a mixing system (according to [19]), where four heating zones were maintained at 170 °C and the screw speed was 200 rpm. The produced pellets were dried (80 °C, 4 h), after which flat films were extruded from them using the Brabender single-screw extruder (Duisburg, Germany) with temperatures of 150–170 °C in three heating zones, 175 °C in the head and with a screw speed of 90 rpm. Films of 100–200 µm thickness were obtained, from which test samples were cut.

### 2.3. Polarization of Composites

Copper electrodes in the form of stick-on copper tapes (type 3M-1181, 50.8 mm wide) were attached to both sides of the samples and placed between the electrodes of the polarizing device. When the polarization temperature *T*_p_ = 90 ± 2 °C was reached, a DC voltage *U*_p_ = 5.3 kV was switched on. Various polarization times were applied: *t*_p_ = {0.25; 0.75; 1; 20} h.

After cooling the sample to 28 ± 2 °C, the voltage was disconnected. Then, 11 mm diameter disks were cut from such polarized samples of 70 mm× 120 mm for piezoelectric testing. For temporal stability evaluation, selected disks were stored under ambient conditions (25 ± 2 °C, 45 ± 2% RH) and re-measured after 30 days.

### 2.4. Thermal, Mechanical, Electrical, and Biological Characterization Methods

#### 2.4.1. Thermogravimetric Analysis (TGA)

Thermogravimetric (TG) analysis was carried out using a thermogravimetric analyzer, model TGA Q200 (TA Instruments, New Castle, DE, USA), in the temperature range from 25 °C to 700 °C, under a nitrogen flow, at a heating rate of 10 °C/min. Samples ranging from 7.8 mg to 10.3 mg were used, which were placed on a platinum pan.

#### 2.4.2. Scanning Electron Microscopy (SEM)

The geometric structure in the cross-section of the extrudate and the dispersion of the BT filler in the polymer matrix were analyzed using scanning electron microscope SU8010 (Hitachi, Tokyo, Japan) using a magnification of ×2000 at an accelerating voltage of 30 kV.

#### 2.4.3. Mechanical Testing (Tensile Properties)

Elongation at break (*ε*_B_), stress at break (*σ*_B_), and tensile strength (*σ*_M_) were determined using a testing machine, model TIRAtest 27025 (TIRA, Schalkau, Germany). The test was conducted in accordance with ISO 527-3: 2019 [38], using a tensile speed of 1.0 mm/min. Specimens cut from sheets of film were clamped in the grips of the testing machine to obtain an initial specimen length (*l*_0_) of—50 mm. Each material variant was tested using *n* = 10 samples.

#### 2.4.4. Dynamic Mechanical Analysis (DMA)

The thermomechanical properties (DMA) of pure PLA films and extruded composites were analyzed using a dynamic mechanical analyzer, model DMA Q800 (TA Instruments, New Castle, DE, USA) in tensile mode at a heating rate of 3 °C/min to 160 °C in an air atmosphere at 1 Hz. Rectangular specimens with dimensions of 12.9 mm × 5.3 mm and thicknesses ranging from 0.14 mm to 0.19 mm were used for the test.

#### 2.4.5. Differential Scanning Calorimetry (DSC)

Differential scanning calorimetry (DSC) measurements were carried out using a differential scanning calorimeter, model DSC Q500 (TA Instruments, New Castle, DE, USA), under a nitrogen atmosphere. Samples of 8 mg to 9.2 mg were used, which were cut from polymer pellets and placed on an aluminum pan for measurements. DSC curves in the temperature range from 0 °C to 210 °C were recorded for three cycles: first heating (10 °C/min), cooling (10 °C/min), and second heating (10 °C/min), maintaining the marginal temperatures for one minute. In order to eliminate the thermal history of the samples, the measurement results were analyzed based on the data from the second heating. The crystallization temperature (*T_c)_*, enthalpy of cold crystallization (*H_cc_*), melting temperature (*T_m_*), and enthalpy of melting (*H_m_*) were determined.

#### 2.4.6. Piezoelectric Coefficient Measurement (d_33_)

The piezoelectric coefficient *d*_33_ was measured on specimens of 11 mm diameter and 150–200 µm thickness with pasted copper electrodes (3M-1181-12) using the ‘Berlincourt’ method using the PM200 PiezoMeter System by Piezotest (Piezotest, Singapore). The measurements were carried out under the conditions recommended by the manufacturer, i.e., frequency 110 Hz, dynamic force of 0.25 N, static force (initial pressure) of 12.0 N. Measurements were carried out under the following conditions: humidity: (45 ± 2)%, ambient temperature (25 ± 2) °C. The presented measurement results are the average value determined for 10 samples.

#### 2.4.7. Dielectric Permittivity Measurements (ε′)

The relative permeability ε_w_ was measured in accordance with PN-86/E-04403 in a three-electrode arrangement. The diameter of the sample was 25 mm, the diameter of the measuring electrode was 10 mm, and the gap between the measuring and protective electrodes was 2 mm. The samples were placed in a Faraday cage, and the values were recorded using an HM8118 LCR impedance bridge in the 0.02–50 kHz range. Measurements were carried out under the following conditions: humidity: (45 ± 2)%, ambient temperature (21 ± 1) °C.

#### 2.4.8. Biodegradation Assessment (ISO 846)

The biodegradation potential of the material was evaluated according to Method A of ISO 846 [39], which is commonly used to test the resistance of polymeric materials to mold. The samples used in this test were not polarized. This method consists of evaluating the growth of mold fungi on the surface of polymer samples incubated in a mineral substrate devoid of carbon sources, which allows them to grow only if the tested material is used as a substrate. For each material variant, three independent samples (*n* = 3) were tested. The obtained results allow for a preliminary selection of samples showing biodegradation potential and their qualification for further studies verifying the polymer biodegradation process.

The samples were incubated in the presence of a suspension of fungal spores. The following model strains of mold fungi were used in the study: *Aspergillus niger* (ATCC 6275), *Penicillium pinophilum* (ATCC 36839), *Paecilomyces variotii* (ATCC 18502), *Trichoderma virens* (ATCC 9645), *Chaetomium globosum* (ATCC 6205). Bacteria usually multiply faster than fungi, but on polymer surfaces, it is the fungi that colonize the samples more visibly [40,41]. Thus, their growth can be more easily assessed during a 28-day incubation under laboratory conditions [42]. To prepare the inoculum for further biodegradation studies, fungal strains were grown on Potato Dextrose Agar (PDA, Oxoid, Basingstoke, Hampshire, UK) at 28–30 °C until the agar surface was completely covered by mycelia. Once the appropriate growth phase was achieved, the fungi were resuspended in sterile saline solution and used to inoculate polymer samples in ISO 846-compliant tests [39].

Visual evaluation of the samples was carried out with the unaided eye according to the guidelines of the standard, using a template with sectors of 5 mm × 5 mm. Based on Table 2, the percentage of overgrowth of the sample surface was evaluated.

Photographic documentation of the samples was also made, and SEM was used to analyze the structural changes of the surfaces in detail. Sample sections measuring 20 × 20 mm were prepared for analysis. Before imaging, the samples were coated with a layer of gold (1 nm) using a Cressington Sputter Coater 108 auto (Cressington Scientific Instruments Ltd., Watford, UK) with a thickness control module (Cressington Thickness Monitor MTM-10, Cressington Scientific Instruments Ltd., UK). Imaging was performed at ×1000 magnification using an accelerating voltage of 2 kV.

Weight loss was determined according to the ISO 846 procedure [39]. After incubation, the samples were cleaned according to the ISO 846 procedure [39].

After drying, the samples were weighed on an analytical balance (Mettler Toledo MSI05DU, Mettler-Toledo GmbH, Greifensee, Switzerland) with an accuracy of 0.001 g under controlled humidity and temperature conditions.

Weight loss of samples (%) was calculated according to Equation (1), where the following applies:

(1) $$mass\;loss=\frac{m_{0}-m_{1}}{m_{0}}\times \;100\%$$

$m_{0}$—weight of sample before incubation (g),

$m_{1}$—sample weight after incubation (g).

## 3. Results and Discussion

### 3.1. Thermogravimetric Analysis

**Figure 1:**
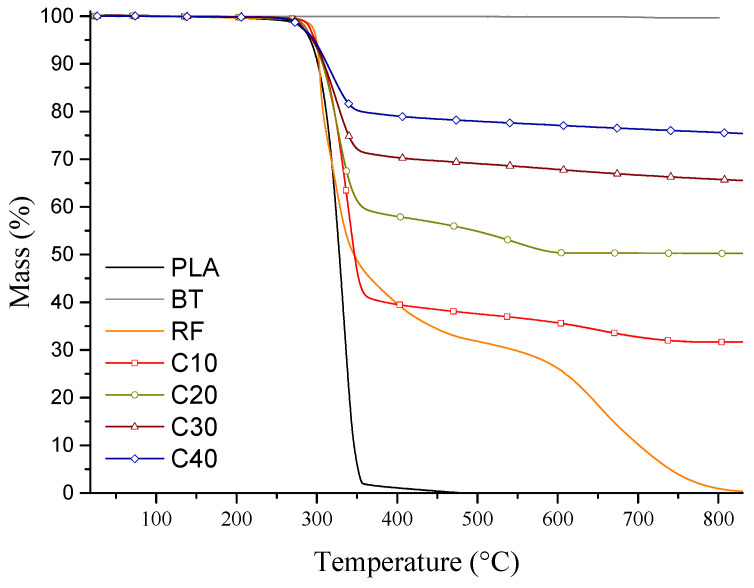
TG curves for PLA, BT, RF, and PLA/BT/RF composites.

Thermogravimetric (TG) analysis was carried out to assess the thermal stability of the PLA/BT/RF composites and to verify the actual filler content. The analysis also made it possible to evaluate the potential impact of riboflavin on the degradation onset of the composite. The TG curves and data are presented in Figure 1 and Table 3 and Table 4.

The TG curves show that BT is thermally stable up to 800 °C, confirming that there is no significant mass loss in this temperature range. In the case of PLA, thermal decomposition starts around 242 °C, reaches a maximum at 334 °C, and ends at 371 °C. RF degraded in two stages, with the first stage (267–379 °C) partially overlapping the PLA decomposition range, making it difficult to accurately determine its content in the composite [32,43].

The actual amount of BT in the sample was determined by the weight of the residue (*M*_p_) after complete degradation of the organic components. The results coincided with the theoretical values (Table 3), and the observed deviations were within acceptable limits for the manufacturing method used.

The introduction of BT and RF affected the thermal stability of the composites. The temperature of onset of decomposition decreased with increasing BT, from 255 °C for C10 to 221 °C for C40, which was due to the lower amount of PLA in the matrix. The values of T_5%_ (the temperature at which weight loss is 5%) were similar in all samples (294.51–297.85 °C), suggesting a similar course of initial degradation.

The results obtained confirm that the actual BT content in the composites is consistent with the theoretical assumptions, and that the applied processing method ensured reproducibility within an acceptable range. The introduction of BT and RF slightly reduced the onset temperature of degradation, especially in composites with high filler content. However, the observed reduction in decomposition onset temperature does not interfere with the processing conditions used in this study (extrusion at 150–175 °C, polarization at 90 °C), which remain well below the degradation thresholds of the components.

### 3.2. Microscopic Analysis

**Figure 2:**
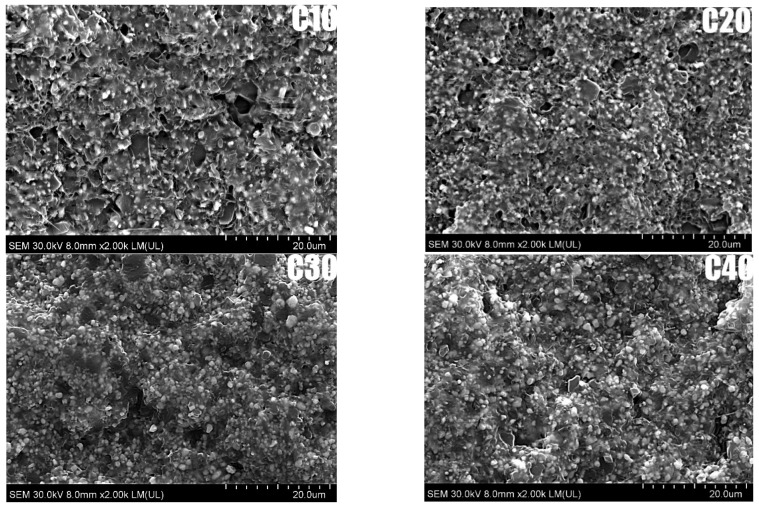
SEM images of composites: C10, C20, C30, and C40.

SEM analysis made it possible to evaluate the microstructure and dispersion of BT particles in the PLA matrix for the composites studied, as shown in Figure 2. SEM analysis showed that BT particles are homogeneously distributed in the PLA matrix, with no obvious signs of agglomeration. The voids are irregularly shaped and randomly distributed, indicating that they are due to mechanical breakage of the sample rather than poor adhesion between matrix and filler. SEM analysis showed good dispersion of BT in PLA for all samples. In the C10 sample, BT particles were evenly distributed, while in C40, a local increase in filler concentration was observed, still without agglomeration.

Importantly, the addition of riboflavin did not disturb the homogeneous distribution of barium titanate in the PLA matrix, confirming the effectiveness of the applied manufacturing method. Uniform ceramic dispersion is essential for achieving a stable piezoelectric response, as it ensures continuous polarization pathways and consistent charge generation under mechanical stress—key factors for reliable performance in biomedical applications.

### 3.3. Mechanical and Thermomechanical Properties

**Figure 3:**
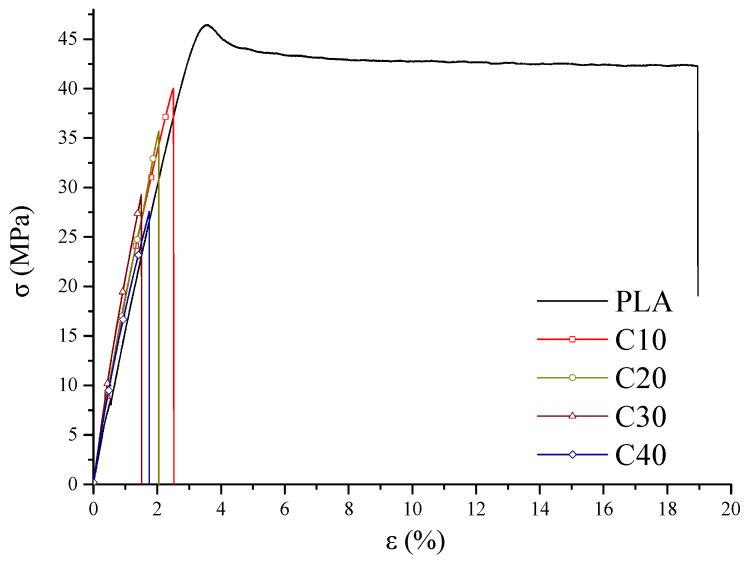
Tensile strength of PLA and PLA/BT/RF composites.

**Figure 4:**
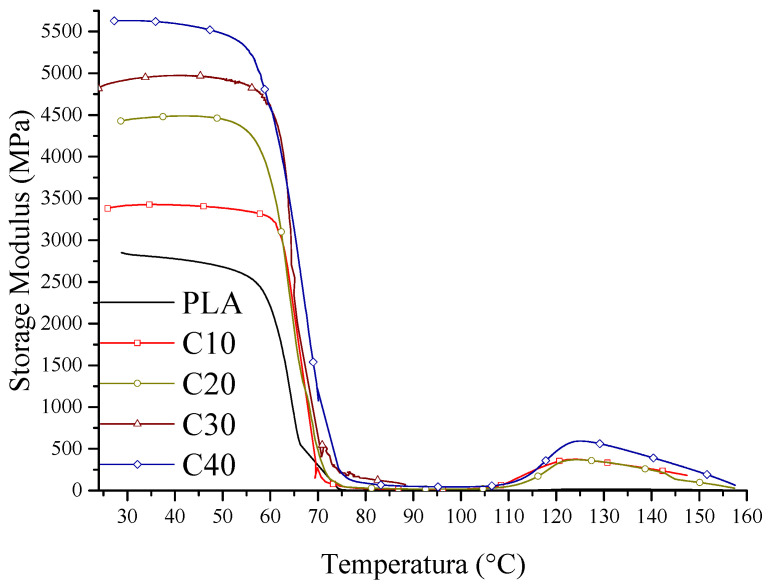
PLA/BT/RF composite storage modulus.

**Figure 5:**
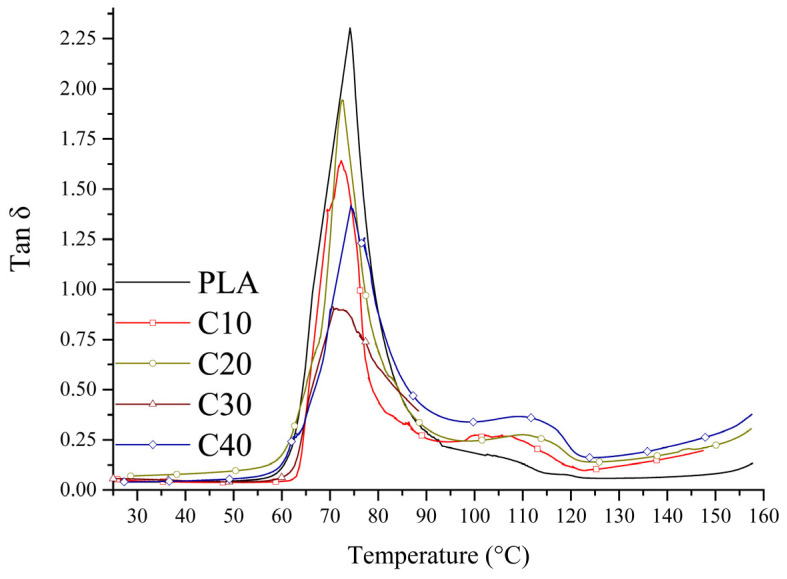
Loss factor of PLA and PLA/BT/RF composites.

The mechanical properties of the tested composites were determined by tensile strength tests and dynamic mechanical analysis (DMA). The results of stress and strain are shown in Figure 3, the values of storage modulus in Figure 4, and the loss factor in Figure 5. Detailed figures are collected in Table 5. This mechanical characterization aimed to evaluate whether PLA/BT/RF composites retain sufficient stiffness and strength for bone tissue engineering, despite the addition of riboflavin intended to promote biodegradation.

As the BT content of the composites increases, a decrease in maximum stress (*σ*_M_) and stress at break (*σ*_B_) is observed, accompanied by increased stiffness of the material. Despite the decrease in tensile strength from 46.5 MPa (PLA) to 27.6 MPa (C40), the material retains adequate stiffness and mechanical resistance for potential biomedical applications, where structural rigidity is important. This tensile strength falls within the range typically reported for biodegradable PLA-based materials used in biomedical applications, including cardiac scaffolds (15–22 MPa), orthopedic implants (up to 42 MPa), and soft-tissue constructs (27–34 MPa) [44,45,46,47,48,49]. In order to determine whether the differences between the PLA/BT and PLA/BT/RF samples at the same BT concentrations are statistically significant, a two-tailed *t*-test for independent means was applied to the mechanical parameters (*σ*_M_, *σ*_B_, *ε*_M_, *ε*_B_, and *T*). Calculated *p*-values showed statistically significant differences (*p* < 0.05) in σM for composites C10–C30 and in *σ*_B_ for C20–C30, while no significant differences in σB were found for C10 and C40 compared to the corresponding V-series. Values with *p* < 0.05 are highlighted in bold in Table 5. The reduction in stress values is noticeable but remains within acceptable limits for biomedical applications where stiffness and dimensional stability are key. This effect is due to a reduction in the mobility of polymer chains by BT particles, which reduces the ability to absorb stress. The calculated toughness (*T*) decreased with increasing BT and RF contents. Statistically significant differences were confirmed between the C- and V-series samples, indicating a consistent reduction in energy absorption due to the RF addition. Similarly, the values of elongation at maximum stress (*ε*_M_) and relative elongation at break (*ε*_B_) were also reduced, with the highest values recorded for PLA and C10, and the lowest for C30 and C40. Statistically significant differences in *ε*_M_ and *ε*_B_ were observed between C10 and V10, and between C40 and V40, whereas other comparisons did not show significant changes.

The increase in the stiffness of the PLA/BT/RF composites is also confirmed by the results of DMA analysis. The storage modulus (*E’*) increases with increasing BT content, which implies greater resistance of the material to elastic deformation. The increased stiffness is due to the reduced mobility of the polymer segments by the stiff BT particles, which take up part of the stresses and reduce the possibility of matrix deformation. This effect is most pronounced in the case of sample C40, demonstrating the effective stiffening of the structure due to the high filler content (Figure 4).

Analysis of the curves (Figure 3) showed that the composites with lower BT content (C10, C20) exhibit plastic deformation capacity before rupture, indicating their greater ability to absorb strain energy. In contrast, in the C30 and C40 samples, rupture occurs suddenly, indicating a transition from plastic to brittle behavior [50,51]. The decrease in loss factor (*tan δ*) with increasing BT content confirms the increasing stiffness of the composite and the limited ability to dampen strain energy (Figure 5).

The glass transition temperatures (*T*_g_) of the tested composites do not show significant changes relative to PLA, indicating that the presence of fillers does not significantly affect this parameter.

A comparison of PLA/BT/RF with PLA/BT [19] showed that the *σ*_M_ and *σ*_B_ values in the RF composites are lower, but the decrease in mechanical strength is not significant enough to rule out their potential use in biomaterials, and the increased stiffness of the material may promote bone tissue deposition. At the same time, despite the decrease in stress, the PLA/BT/RF composites still exhibit the deformation capacity necessary for the piezoelectric effect to occur. The combination of these properties may be beneficial in the context of regenerative engineering, where it promotes tissue regeneration processes [52,53].

The obtained values of elastic modulus (~5.6 GPa) for the PLA/BT and PLA/BT/RF composites clearly exceed typical stiffness values of biodegradable polymers used in implantology, such as PLA (2–3 GPa), PLGA (up to 2.5 GPa), or PCL (0.4–0.6 GPa) [54,55]. Similar stiffness levels in PLA/Mg systems are usually achieved using more complex processing techniques [56]. In contrast, the tested composites combine simplicity of composition, processability, biodegradability, and high rigidity, making them promising for bone tissue engineering.

### 3.4. Crystallization

**Figure 6:**
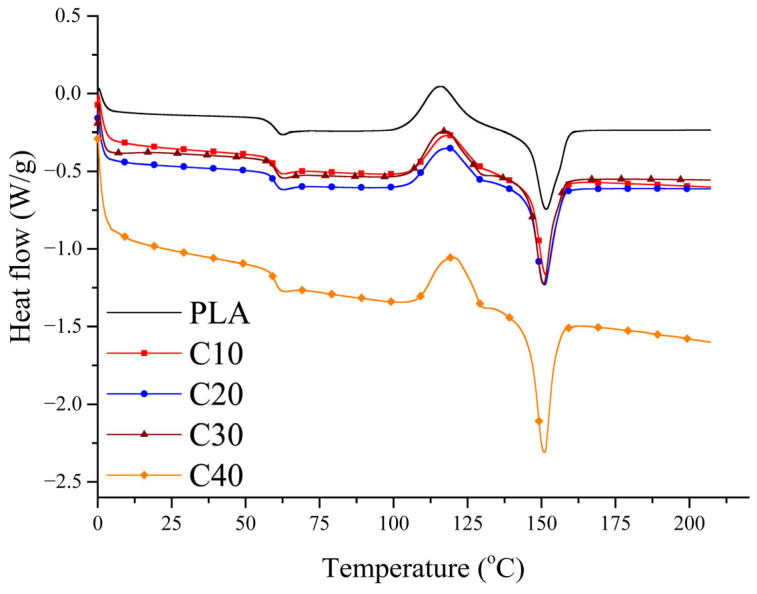
DSC thermograms recorded during the second heating for PLA/BT/RF composites.

The aim of the DSC analysis was to assess whether BT and RF influence the crystallization behavior of PLA, as the degree of crystallinity may affect the stiffness and degradation rate of the composites, which are relevant for biomedical applications. Second heating curves for the composites are shown in Figure 6. The values of glass transition (*T*_g_), cold crystallization (*T*_cc_), and melting (*T*_m_) temperatures, as well as enthalpies of melting (*ΔH*_m)_ and cold crystallization (*ΔH*_cc_) and degree of crystallinity (*χ*_c_), are summarized in Table 6.

All samples tested show PLA’s characteristic phase transitions, confirming that the introduction of BT and RF does not cause radical changes in their thermal properties. The glass transition temperature (*T*_g_) and cold crystallization temperature (*T*_cc_) remain at similar levels in all samples, suggesting that both BT and RF show no significant effect on these parameters.

The melting point (*T*_m_) also remains similar to that of pure PLA, although it is slightly lower in the PLA/BT/RF composites than in PLA/BT, which may be related to the reduced matrix content.

The most prominent difference lies in the degree of crystallinity, which increases with increasing BT content. This indicates that the filler can act as a nucleation center, facilitating the formation of PLA’s crystalline phase. To calculate the degree of crystallinity ($\chi_{c}$) of the composites, the following formula was used, according to Equation (2):

(2) $$\chi_{c}=\frac{∆H}{∆H_{m}^{o}\cdot W_{PLA}}\cdot 100\%$$

where ∆H is the enthalpy of melting, $∆H_{m}^{o}$ is the enthalpy of the melting of 100% crystalline PLA (93 J/g) [57], and $W_{PLA}$ is the mass fraction of PLA in the composite sample

The degree of crystallinity increases with BT content. For pure PLA, it is 27.6%, while in the C40 composite, it reaches 34.9%.

A comparison of the analysis results for PLA/BT and PLA/BT/RF indicates that RF does not significantly affect the glass transition temperature (*T*_g_) or the cold crystallization temperature (*T*_cc_), which remain at similar levels in both composites. The melting temperature (*T*_m_) for PLA/BT/RF is slightly lower than for PLA/BT. The values of the enthalpy of melting (*ΔH*_m_) are similar, while the degree of crystallinity (χ_c_) reaches 33.3% in V40 and 36.7% in C40, which may suggest that the presence of RF promotes an increase in the degree of crystallinity of the composites [19]. An increase in crystallinity is beneficial as it enhances the stiffness of the material, which may contribute to improved mechanical stability and polarization efficiency. However, higher crystallinity can also reduce the biodegradability of PLA, which must be considered in biomedical applications.

The addition of RF increased the crystallinity (χc) of the PLA/BT composites by approximately 8%, without significantly altering the glass transition temperature (*T*_g_), the cold crystallization temperature (*T*_cc_), or the microstructure of the material. A statistically significant decrease in tensile strength (σ_M_) was observed for the C10–C30 composites, accompanied by a significant increase in elongation at break (ε_B_) for C40 compared to V40, suggesting slightly improved ductility within this group. The decomposition temperature was reduced by approximately 30 °C due to the presence of the organic additive; however, this change did not impair the processing, poling efficiency, or functional usability of the composite, confirming its suitability for technical applications.

### 3.5. Electrical and Piezoelectric Properties

The PLA/BT/RF composite exhibits piezoelectric properties. The value of the piezoelectric coefficient *d*_33_ depends on BT content and polarization time, as shown in Table 7.

As for the PLA/BT composite, for the PLA/BT/RF composite, the value of the piezoelectric coefficient *d*_33_ increases with higher BT content and with longer polarization time [58]. So, the value of the piezoelectric coefficient is highly dependent on the polarization of the filler [58,59]. It should also be noted that due to the comparison with the previously described PLA/BT composite, the polarization process was carried out under the same conditions and was not optimized. Optimization of the polarization process is complex [58] and needs further research, especially in connection with the development of piezocomposites. The piezoelectric response was almost symmetrical, with similar d_33_ values on both sides of the sample, falling within the range of measurement uncertainty. For determining whether the differences between the groups PLA/BT/RF and PLA/BT for the same BT concentrations and polarization times occur, the *t*-test for two independent means calculations was applied. The calculated *p*-value (two-tailed test) showed that the null hypothesis cannot be rejected due to a lack of statistical significance for some samples, where *p*-value > 0.05. That situation occurs for the PLA/BT/RF and PLA/BT pairs highlighted (bold) in Table 7, where only the V20/C30 samples (polarization time 0.25 h, neg.) and V10/C10 (polarization time 0.45 h, neg., pos.) show an increase in mean value of piezoelectric coefficient with the RF addition. In other cases, the value of the piezoelectric coefficient *d*_33_ decreased by about 10–30% depending on the polarization time. The highest decrease was observed for samples with the highest BT concentration, i.e., C40. Despite some reduction in d_33_ values upon the RF addition, all measured values remained within the range considered sufficient to stimulate osteogenic activity.

To verify the temporal stability of the piezoelectric response, sample C40 was stored under ambient conditions (25 ± 2 °C, 45 ± 2% RH) and re-measured after 30 days. The piezoelectric coefficient d_33_ was determined again, and the results are presented in Table 8.

After 30 days of ambient storage, the piezoelectric coefficient decreased by 9.6% for the sample polarized for 1 h and by 8.5% for the sample polarized for 20 h. The observed decline indicates only partial retention of piezoelectric properties and suggests that polarization conditions may require further optimization. These findings underline the need for extended research on long-term functional stability under physiological degradation.

**Figure 7:**
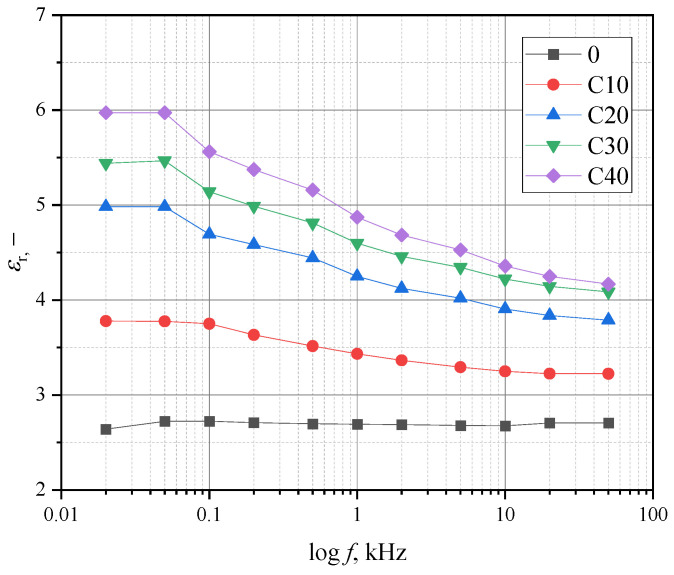
Frequency dependence of permeability for different concentrations.

Figure 7 shows the relationship between relative frequency and permeability.

The decrease in permeability as a function of frequency is as follows: 15, 24, 25, and 30% for the samples with BT content of 10, 20, 30, and 40%, respectively. For the samples without the RF addition, the decrease was barely observable, amounting to a few percent.

The addition of RF also reduced the electrical permeability values relative to the PLA/BT composite. For the PLA/BT/RF composite tested, it is in the range of 3.5 to 5.5, which is lower than the values measured for bone [60]. Increasing the permeability is possible and can be realized using appropriate models for dielectric mixtures [61].

Table 9 shows the results of measuring the relative permeability and piezoelectric voltage constant *g*_33_ for a frequency of 1000 Hz. To determine the values of the *g*_33_ coefficient, the values of the piezoelectric coefficient *d*_33_ with negative polarity were taken for a polarization time of 20 h. The permeability values are smaller than those of the PLA/BT composite. The addition of RF reduced the relative permeability by up to 50% for samples with a high concentration of BT (C30 and C40). Nevertheless, the *g*_33_ factor is comparable to or even higher than for typical ceramic materials and ceramic composites [62,63,64,65].

Although the addition of RF caused a moderate decrease in d_33_ at higher BT loadings, the piezoelectric effect remained present [66]. Even in the C40 composite, with the highest BT and RF contents, the *d*_33_ value (3.92 pC/N) remained above the osteogenic threshold (~2 pC/N) [60,61], confirming the clinical suitability of PLA/BT/RF materials [14,25].

### 3.6. Biodegradability

**Figure 8:**
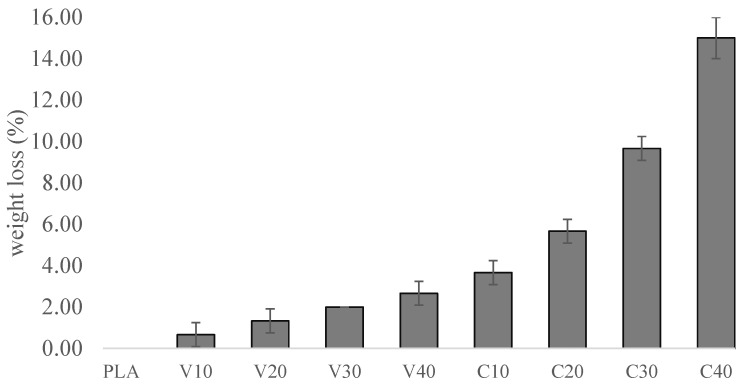
Weight loss of samples after incubation.

To quantitatively assess the biodegradation of the PLA/BT and PLA/BT/RF composites, an analysis of the weight loss of the samples after a period of incubation in a fungal suspension was performed. The results are shown in Figure 8, which allowed the effect of both BT and RF alone on the degradation of the materials to be determined.

The PLA/BT samples showed low weight loss (about 1 to 2%), which did not change significantly with increasing BT content, suggesting that the degree of filling does not significantly affect composite degradation.

The PLA/BT/RF samples had significantly higher weight loss compared to the composites containing BT alone. Increased filler content resulted in a systematic increase in weight loss, reaching a maximum of 16% for the C40 samples.

The results of the mass loss analysis are consistent with the results of other studies evaluating the biodegradation of composites. The high mass loss in the PLA/BT/RF samples indicates more intensive degradation of the material, which was reflected in the degree of surface coverage by microorganisms [42,67,68]. This was confirmed by visual analysis of the samples, the results of which are presented below.

**Figure 9:**
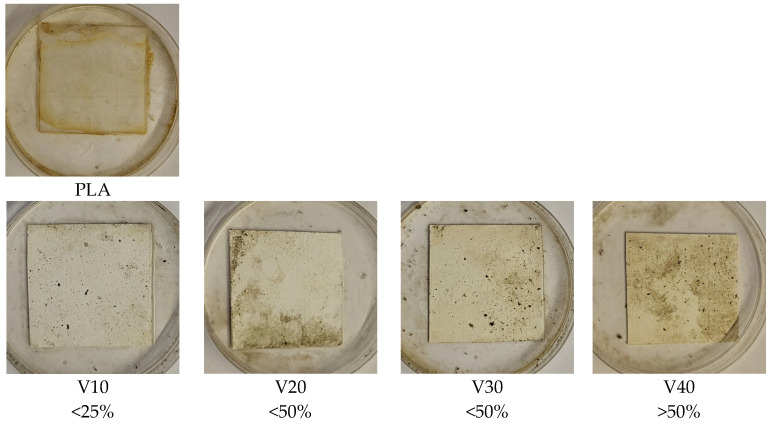

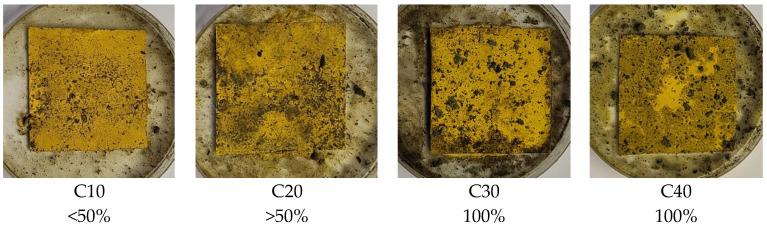
Visual analysis of samples after incubation in a fungal suspension, with the approximate area of microbial colonization of samples determined by the scale adopted in ISO 846 [39].

Visual evaluation of the surface of the samples after the incubation period (Figure 9) showed clear differences in the degree of microbial colonization depending on the filler content. The PLA/BT composites showed limited susceptibility to colonization by microorganisms, as confirmed by the low degree of surface coverage by mycelium [69,70]. The highest colonization in this group was observed in the V40 sample and, according to the accepted evaluation scheme (Table 2), was in the range corresponding to more than 50% of the surface area, indicating that even at the highest BT content, microbial growth remained limited.

The presence of RF promoted microbial colonization, evidenced by more extensive mycelial growth on the sample surfaces. In the PLA/BT/RF composites, full mycelial coverage (100%) was observed in C30 and C40, confirming the increased susceptibility to biological degradation. This effect is attributed to riboflavin’s biological role as a cofactor in the TCA cycle, which supports fungal metabolism and colonization. The results are consistent with previous findings, showing that RF enhances microbial adhesion, followed by biofilm formation and subsequent degradation [19,71].

While the PLA/BT composites showed only a slight susceptibility to biodegradation, the addition of RF significantly intensified the process. While the samples containing BT alone remained relatively resistant to microbial growth, the presence of RF led to their markedly greater colonization by mycelium, clearly confirming its role as an enhancer of biological degradation.

**Figure 10:**
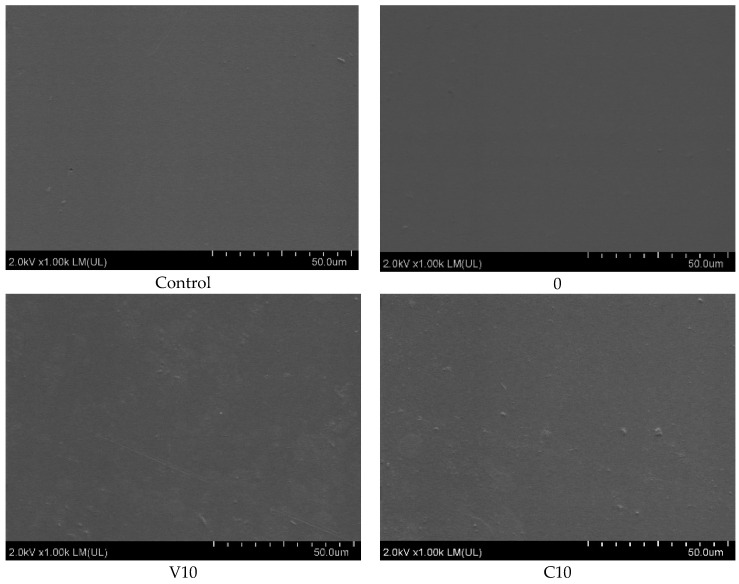

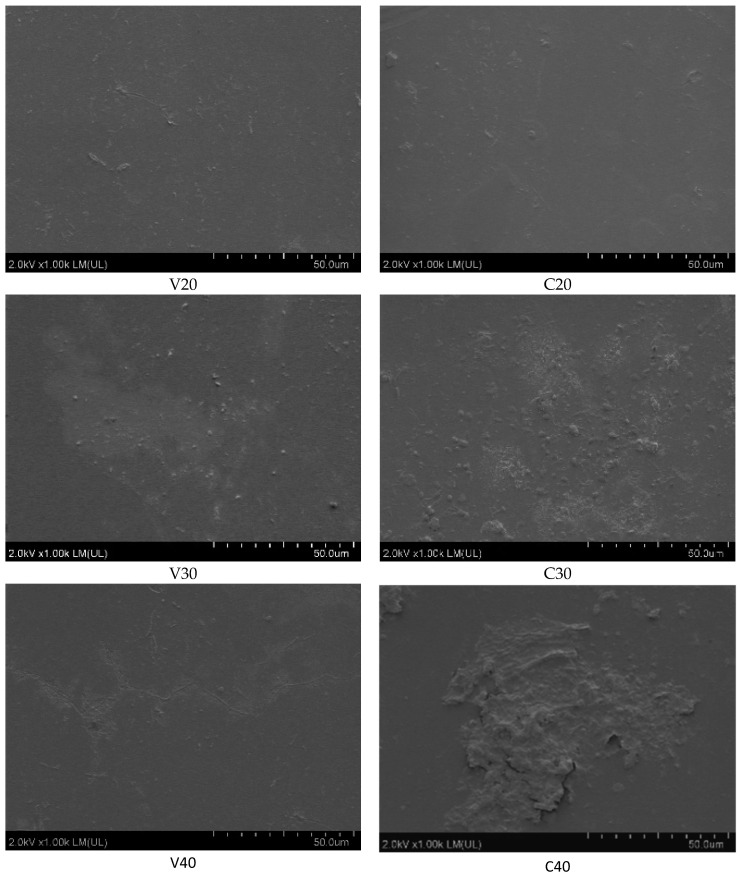
SEM analysis of samples after incubation in fungal suspension.

The observed changes in color and surface structure of the PLA/BT/RF samples after incubation indicated progressive degradation, as confirmed by SEM analysis (Figure 10). As the BT content of PLA/BT increased, a gradual increase in surface changes was observed, from single structures in V10 to filamentous fungal-like forms and circular depressions in V40. Such structures may indicate intense microbial activity, which agrees with previous observations of mass loss. In the C samples, the changes were more advanced, especially in C30 and C40, where numerous thickenings and a cracked, crusted layer of biofilm appeared. In many places, this may have been its remnants, not removed completely during the rinsing according to the ISO 846 procedure [39].

The observed increase in mass loss in the samples containing RF further confirms its role in enhancing the biodegradability of the composite. Riboflavin, as a biologically active molecule, may facilitate fungal colonization, as also indicated by microscopic observations. A likely mechanism involves its function as a precursor of the flavin cofactors FMN and FAD, which participate in the tricarboxylic acid (TCA) cycle and electron transport chain in many microorganisms [72,73,74]. Enhanced availability of these cofactors may stimulate fungal metabolic activity and enzymatic expression—particularly esterases and lipases—contributing to the breakdown of ester bonds in the PLA matrix. Additionally, the amphiphilic nature of RF may support spore adhesion and biofilm formation, further accelerating surface degradation. These effects were observable when comparing the RF-containing composites with their reference counterparts [32].

These findings reinforce the dual-functional role of riboflavin as both a biological activator and a degradation promoter in PLA-based composites.

## 4. Conclusions

This study demonstrates that the addition of riboflavin (RF) significantly enhances the biodegradability of PLA/BT composites. After 28 days of exposure according to ISO 846 [39], the weight loss for PLA/BT composites was about 3%, while for PLA it was close to zero. The addition of RF significantly accelerated biodegradation, as evidenced by the weight loss of the C40 sample at 15%, five times higher than in V40. Increased RF content correlated with more extensive microbial colonization, confirming its role in accelerating the composite’s biodegradation. RF does not significantly affect the thermal and mechanical properties of PLA/BT composites, maintaining their thermal stability (*T*_g_~61–62 °C) and mechanical strength at levels comparable to unmodified PLA/BT (*σ*_M_~27–40 MPa). The presence of RF leads to a decrease in the piezoelectric coefficient *d*_33_, especially in samples with a high BT content, where it decreases by 26% relative to the V40 sample. Additionally, a decrease of the piezoelectric coefficient *d*_33_ of less than 10% was observed after 30 days. However, this reduction remains within the range reported to stimulate osteogenic activity, with the C40 composite reaching approximately 3.9 pC/N [25,75]. Moreover, the value of the piezoelectric coefficient increases with BT content and with polarization time. The polarization process was not optimized and requires further research. SEM analysis confirmed the homogeneous dispersion of BT particles in the PLA matrix, and RF does not disturb the filler distribution in the composite structure.

Combining the piezoelectric effect with controlled biodegradation, PLA/BT/RF composites can find applications in tissue engineering as biodegradable materials, especially to support bone regeneration. The enhanced biodegradation may additionally allow for faster resorption of the implant once it has served its function, which is beneficial for temporary implants to support tissue regeneration. Accelerated degradation due to the RF addition may enable more predictable and timelier implant resorption, better aligned with the healing process. This is particularly beneficial for temporary implants used to support tissue regeneration, as it minimizes the risk of chronic inflammation and the need for secondary surgery. The addition of riboflavin significantly accelerated microbial colonization and mass loss in PLA/BT composites. These effects are likely related to the physicochemical properties of RF and its role in microbial metabolism. While promising for biodegradable implant applications, further in vitro studies are required to assess cytocompatibility under physiological conditions.

## Figures and Tables

**Table 1:** Determination of samples according to filler content.

Sample Name	Ingredient Content		
	PLA (% vol. %)	BT (vol. %)	RF (mas. %)
C10	70	10	20
C20	60	20	20
C30	50	30	20
C40	40	40	20
V10	90	10	-
V20	80	20	-
V30	70	30	-
V40	60	40	-

**Table 2:** Evaluation of microbial growth.

Number of Squares with Growth in Visual Assessment	Evaluation
0	No visible growth under the microscope
0	Growth invisible to the unaided eye, but clearly visible under a microscope. Covering up to 25% of the sample surface.
0	Growth invisible to the unaided eye, but clearly visible under a microscope. Covering up to 50% of the sample surface.
0	Growth invisible to the unaided eye, but clearly visible under a microscope. Covering more than 50% of the sample surface.
1 to 16	Growth discernible with the unaided eye, covering up to 25% of the study area.
17 to 32	Growth discernible with the unaided eye, covering up to 50% of the study area.
33 to 64	Significant growth, covering more than 50% of the study area.
above 64	Intense growth covering the entire study area (only if the edge influence is particularly significant).

**Table 3:** Assumed and thermogravimetrically determined filler content in the composite.

Sample Name	BT Content (wt%)	
	Theoretical	Designated
C10	33.67	31.55
C20	53.10	50.23
C30	65.75	65.74
C40	74.65	75.05

**Table 4:** Summary of TG analysis results.

Sample Name	Decomposition Onset Temp (°C)	Max Peak (°C)	End of Decomposition Temp (°C)	T_5%_ (°C)	M_p_ (%)
PLA	241.77	334.41	371.51	291.87	0.11
RF	266.82	266.82	378.64	304.30	0.99
C10	254.96	353.82	379.37	297.85	31.55
C20	245.72	362.34	373.59	294.51	50.23
C30	235.41	363.75	371.97	294.81	65.74
C40	221.49	339.54	365.53	296.53	75.05

**Table 5:** Tensile strength (*σ*_M_), stress at break (*σ*_B_), elongation at maximum stress (*ε*_M_), elongation at break (*ε*_B_), and toughness (*T*) determined for the tested samples.

Sample	*σ*_M_ (MPa)	*σ*_B_ (MPa)	*ε*_M_ (%)	*ε*_B_ (%)	*T* (kJ/m^3^)
PLA	46.46 ± 2.89	42.30 ± 2.58	3.53 ± 0.28	18.95 ± 0.28	1797 ± 110
C10	**40.04 ± 4.09**	40.04 ± 4.56	**2.51 ± 0.28**	**2.51 ± 1.02**	**32 ± 3.50**
C20	**35.72 ± 3.34**	**35.72 ± 3.34**	2.05 ± 0.23	2.05 ± 0.23	**21 ± 2.00**
C30	**29.31 ± 5.51**	**29.31 ± 5.51**	1.50 ± 0.29	1.50 ± 0.29	**11 ± 2.00**
C40	27.59 ± 4.17	27.59 ± 4.18	**1.75 ± 0.34**	**1.75 ± 0.34**	**15 ± 3.00**

**Table 6:** Summary of numerical values of temperatures and enthalpies for PLA and composite phase transitions.

Sample	*Tg* (°C)	*Tcc* (°C)	*Tm* (°C)	Δ*Hm* (J/g)	*ΔHcc* (J/g)	*χc* (%)
PLA	62.0	116.0	145.8	26.9	26.7	28.9
C10	60.9	119.0	151.6	27.3	26.2	29.3
C20	61.3	118.6	145.9	26.3	26.1	28.3
C30	61.0	117.6	150.7	29.3	26.2	31.5
C40	61.5	120.2	145.8	34.1	30.4	36.7

**Table 7:** Effect of polarization time on piezoelectric coefficient *d*_33_ for samples with different BT contents.

Samples	Polarity	Polarization Time, h			
		20	1	0.75	0.25
C40	neg.	3.92 ± 0.21	3.88 ± 0.57	3.13 ± 0.12	2.52 ± 0.10
	pos.	4.22 ± 0.81	3.39 ± 0.10	3.64 ± 0.32	2.58 ± 0.12
V40	neg.	5.28 ± 0.29	4.82 ± 0.39	-	-
	pos.	5.14 ± 0.18	4.60 ± 0.17	-	-
C30	neg.	3.13 ± 0.12	2.66 ± 0.07	2.27 ± 0.08	**1.49 ± 0.06**
	pos.	3.53 ± 0.18	2.71 ± 0.07	2.46 ± 0.13	1.46 ± 0.05
V30	neg.	4.67 ± 0.37	3.39 ± 0.26	2.95 ± 0.06	**1.25 ± 0.14**
	pos.	4.49 ± 0.34	3.30 ± 0.24	2.95 ± 0.06	1.28 ± 0.11
C20	neg.	**2.01 ± 0.14**	**1.86 ± 0.08**	**1.49 ± 0.09**	0.79 ± 0.03
	pos.	**1.99 ± 0.45**	**1.88 ± 0.15**	**1.41 ± 0.05**	0.80 ± 0.03
V20	neg.	**2.11 ± 0.06**	**2.08 ± 0.22**	**1.36 ± 0.32**	0.57 ± 0.02
	pos.	**2.17 ± 0.07**	**2.51 ± 0.34**	**1.43 ± 0.33**	0.55 ± 0.05
C10	neg.	0.69 ± 0.03	0.62 ± 0.03	**0.57 ± 0.02**	0.51 ± 0.01
	pos.	0.70 ± 0.03	0.67 ± 0.05	**0.56 ± 0.02**	0.51 ± 0.02
V10	neg.	0.35 ± 0.07	0.33 ± 0.02	**0.81 ± 0.13**	0.15 ± 0.06
	pos.	0.44 ± 0.03	0.38 ± 0.02	**0.75 ± 0.08**	0.13 ± 0.05
0	neg.	0.05 ± 0.03	0.08 ± 0.03	0.38 ± 0.08	0.04 ± 0.02
	pos.	0.13 ± 0.06	0.22 ± 0.06	0.29 ± 0.06	0.07 ± 0.02

**Table 8:** Piezoelectric coefficient d_33_ of PLA/BT composites containing 40 vol% BT, measured after different polarization times and after 30 days of ambient storage to evaluate temporal stability.

Time After Polarization, Days	Time of Polarization, h	
	1	20
	Piezoelectric Coefficient *d*_33_, pC/N	
7	3.88 ± 0,57	3.92 ± 0.21
30	3.51 ± 0,52	3.59 ± 0.19
Percentage decrease, %	9.6	8.5

**Table 9:** Relative permeability of samples for measurement frequency of 1000 Hz, with the value of piezoelectric coefficient *d*_33_ adopted for calculation of piezoelectric voltage constant *g*_33._

Sample	0	C10	V10	C20	V20	C30	V30	C40	V40
*ε*_w_, -	2.69	3.43	4.32	4.12	4.53	4.86	7.16	5.37	8.20
*d*_33_, pC/N	0.05	0.69	0.35	2.01	2.11	3.13	4.67	3.92	5.28
*g*_33_, ×10^−3^ Vm/N	2.10	22.7	9.20	55.1	52.6	72.8	73.7	82.5	72.8

## Data Availability

The original contributions presented in this study are included in the article material. Further inquiries can be directed to the corresponding authors.
